# Evaluation of the Relationship Between Trace Element Levels and Cellular Adhesion Molecules (ICAM-1, VCAM-1) in Hemodialysis Patients

**DOI:** 10.3390/jcm15051979

**Published:** 2026-03-05

**Authors:** Duygu Felek, Mustafa Fatih Erkoc, Kubra Kurul, Vugar Ali Turksoy

**Affiliations:** 1 Department of Internal Medicine, Faculty of Medicine, Yozgat Bozok University, Yozgat 66100, Turkey; 2 Department of Radiology, Faculty of Medicine, Yozgat Bozok University, Yozgat 66100, Turkey; mustafafatih.erkoc@bozok.edu.tr; 3 Department of Internal Medicine, Yozgat Sorgun State Hospital, Yozgat 66000, Turkey; 4 Department Of Public Health, Faculty Of Medicine, Yozgat Bozok University, Yozgat 66100, Turkey

**Keywords:** haemodialysis, trace element, ICAM-1, VCAM-1

## Abstract

**Background**: Both chronic kidney disease (CKD) and the haemodialysis procedure can contribute to disturbances in mineral homeostasis, which can potentially result in cellular pathologies. Our study aims to investigate trace element levels in haemodialysis patients and evaluate their potential impact on cellular adhesion molecules. This will clarify the clinical significance of trace element imbalances in this population. **Methods**: The study included 84 haemodialysis patients and 42 healthy controls. Trace element levels in blood (Zn, Cu, Mn, Mo, V, Sb and Cr) were measured using inductively coupled plasma mass spectrometry (ICP-MS), and cellular adhesion markers ICAM-1 and VCAM-1 were analysed by ELISA. Data analysis was conducted using SPSS 20.00, with significance set at *p* < 0.005. **Results**: Manganese (Mn) levels were significantly higher in haemodialysis patients (*p* = 0.019). Copper (Cu), Molybdenum (Mo), Vanadium (V), Antimony (Sb) and Chromium (Cr) levels were higher in the control group. Zinc (Zn) and Cr levels differed significantly between the control group (*p* = 0.018; *p* = 0.007). Cu levels were lower in hypertensive patients (*p* = 0.011), while Zn and Mn levels were higher in diabetic patients (*p* = 0.048 and *p* = 0.004, respectively). Dialysis duration, however, correlated with Sb (r = 0.295; *p* = 0.01), and Kt/V correlated with Mn, Sb and Cr (r = 0.256, r = 0.272 and r = 0.259, respectively; *p* = 0.05). Mo levels showed a positive correlation with both pre-dialysis (r = 0.230) and post-dialysis (r = 0.281) creatinine levels, and a negative correlation with post-dialysis GFR (r = −0.294). ICAM-1 and VCAM-1 levels were significantly elevated in dialysis patients (*p* = 0.001 for both); however, it was not found to be related to variables in the vascular access route. **Conclusions**: The levels of trace elements and adhesion molecules were examined in haemodialysis patients. High Mn levels indicate a risk of accumulation, while low Cu, Mo, V, Sb and Cr levels may require monitoring for deficiency. ICAM-1 and VCAM-1 levels in haemodialysis patients are associated with some trace elements (Mn and Zn); however, this relationship requires further evidence. In conclusion, the levels of trace elements and adhesion molecules in haemodialysis patients indicate the need for regular monitoring and show that the relationships between creatinine and GFR can be applied to larger patient groups.

## 1. Introduction

Trace elements are substances found in very low concentrations in the body and can cause various pathologies when deficient or excessive. While they generally do not cause clinical problems in healthy individuals, this balance can be significantly disrupted in individuals with chronic kidney disease (CKD). Therefore, although serum trace element levels are not routinely monitored in clinical practice, they represent an area that should be evaluated in CKD patients, particularly those undergoing haemodialysis (HD). Along with impaired kidney function, meaningful changes in trace element levels can be observed, similar to electrolyte balance; this situation becomes more pronounced in HD patients [1].

Trace elements are present in small amounts in almost all foods found in nature and can be metabolised and excreted without causing pathology in normal physiology. In cases of deficiency, they can be supplemented externally. However, it is known that these elements are associated with numerous pathologies not only due to their primary effects but also because of their roles in many enzymatic reactions and cellular processes [2]. This situation has led to the emergence of a broad field of research, including which patient groups should be evaluated and which clinical findings should raise suspicion. Clinical effects are observed across a broad spectrum. For example, copper deficiency is associated with abnormalities in erythrocyte and leukocyte series, oxidative stress, neurodegenerative pathologies, and multiple organ damage, while copper excess has also been shown to cause neurotoxicity [3]. Similarly, zinc deficiency has been associated with dermal pathologies, atypia, allergic conditions, developmental disorders, and neurological pathologies. Both deficiency and excess of these elements, which are clinically problematic, have also been noted in studies of HD patients. These studies reported that zinc and selenium deficiencies, as well as nickel excess, are common in HD patients and therefore recommended monitoring [4]. Findings supporting the clinical importance of trace element imbalance include studies demonstrating impaired cognitive function, variability in sleep quality, increased risk of coronary artery disease, and association with mortality [5,6,7]. Furthermore, trace elements have been shown to influence the regulation of adhesion molecules (ICAM-1, VCAM-1) at the cellular level and may be associated with vascular inflammation, coronary artery disease, and increased mortality through this pathway. These mechanisms provide important biological links explaining why vascular pathologies are prominent in HD patients [8,9].

The haemodialysis process is based on the physiology of exchange between blood and dialysate, and trace element levels are also affected by this process. Factors such as dialysate composition, membrane properties, and dialysis duration are used to mimic physiological equilibrium as closely as possible [10]. However, studies showing that dialysis-related aluminium toxicity causes haematological disorders have clearly demonstrated the importance of selecting the appropriate fluids and materials [11]. For this reason, dialysates are regularly monitored, and maximum limits are set for the chemical substances they contain. Data published by the American Public Health Association in 1995 defined maximum concentrations for metals such as antimony, arsenic, aluminium, copper, zinc, fluorine, cadmium, chromium, and lead [12]. Today, dialysate production and water system monitoring continue in line with these limits. However, these regulations are primarily aimed at preventing dialysis-related toxicity. Individual assessments based on blood trace element levels are not performed in haemodialysis patients; there is no practice of adding missing elements to the dialysate or reducing excess elements. This situation may lead to subclinical imbalances continuing unnoticed.

Patients with chronic kidney disease and those undergoing haemodialysis are at risk of deficiencies in essential trace elements (e.g., zinc and selenium) and the accumulation of toxic elements (e.g., lead and arsenic). This situation arises due to impaired mineral homeostasis associated with the loss of renal function and dialysis. Systematic reviews have shown that imbalances in trace elements are common in haemodialysis patients. Such imbalances have been linked to clinical pathologies such as oxidative stress, immune system dysfunction, an increased risk of infection and metabolic complications [13,14]. Adhesion molecules, particularly members of the immunoglobulin superfamily such as ICAM-1 and VCAM-1, facilitate leukocyte adhesion to the endothelium during inflammation and maintain the inflammatory response [15,16]. Increased circulating adhesion molecules in patients with chronic kidney disease have been associated with disease stage and indicators of inflammation, suggesting that adhesion molecules may be pathophysiologically important in CKD [17]. Given the role of trace elements in regulating oxidative stress and the inflammatory response, imbalances in these elements are predicted to indirectly affect the adhesion molecules involved in cell-to-cell and cell-to-endothelial interactions.

KDIGO (Kidney Disease: Improving Global Outcomes) dietary guidelines detail the control of macrominerals/oligominerals such as protein, phosphorus, and health status in CKD patients, but do not directly address trace elements; however, they recommend monitoring them. The NKF/KDOQI (National Kidney Foundation/Kidney Disease Outcomes Quality Initiative) guideline contains more specific assessments regarding the dietary preparation of trace elements; it specifies criteria for ensuring adequate dietary intake and supplementation if necessary, rather than a daily intake budget [18]. Since the study recommended monitoring trace element levels in haemodialysis patients according to the KDIGO nutrition guidelines, the aim was to evaluate whether there were differences between groups in terms of both trace elements and cellular adhesion molecules and whether there was a relationship between these elements and cellular adhesion molecules. Additionally, the study aimed to evaluate the potential effects of both trace elements and cellular adhesion molecules, examine how these levels are affected by haemodialysis-related parameters, and reveal their clinical significance.

## 2. Materials and Method

### 2.1. Ethical Approval and Study Design

This study was conducted with the approval of the Yozgat Bozok University Non-Interventional Clinical Research Ethics Committee (approval number 2024-GOKAEK-2415_2024.12.18_217). The study took place at Yozgat Bozok University and included individuals undergoing routine haemodialysis due to chronic renal failure, as well as a control group with healthy kidney function. Those aged 18 years or over, who had been diagnosed with atrophic kidneys via radiological imaging and had experienced kidney dysfunction for over three months, as well as having been on a routine haemodialysis programme for at least six months, were included in the study. The healthy control group comprised individuals over 18 years of age, whose age-related nephron loss did not affect GFR [19], with no history of chronic disease or family history of genetically transmitted kidney disease, who were not taking vitamin and mineral supplements and in whom no pathology had been detected in previous renal imaging. The study included 90 patients undergoing haemodialysis due to chronic kidney failure and 45 healthy controls. Six patients and three healthy controls were excluded due to the presence of metallic foreign bodies, meaning they did not meet the inclusion criteria. Thus, the study included 126 individuals. This cross-sectional observational study included 84 haemodialysis patients and 42 healthy controls. Power analysis (with effect size 0.572 and alpha 0.05 parameters, two-tailed according to the hypothesis) calculated the actual power as 85.15%.

Haemodialysis patients were monitored by a single dialysis physician. The dialysis materials used for haemodialysis were uniform, and the same dialyzer appropriate for the body surface area was used. The water used for dialysis is supplied from the same network and is controlled according to national standards. Calcitriol and calcimimetics associated with secondary and tertiary hyperparathyroidism were administered parenterally in accordance with the KDIGO guidelines based on the iron parameters used by haemodialysis patients. It was observed that a complete CKD diet was not generally followed in CKD patients specific to the study. Water content in both groups did not contain minerals. The principles of the drinking water trace element study conducted in our region were measured within the WHO mineral-free drinking water standard limits. It was evaluated that drinking water is not a primary source of trace elements and that trace element levels can be reduced as long as mineral water is not consumed [20,21].

### 2.2. Data Collection and Analysis

All samples were taken at the start of haemodialysis for testing purposes as part of specific monthly routines in the patient group, and simultaneously in the control group during routine sampling, observing a morning fasting period (at least 8 h). The samples, taken from both the patient and control groups, were centrifuged at 3000 rpm for 10 min in biochemistry tubes. The resulting supernatant was then stored in Eppendorf tubes at −20 °C until analysis. The samples were then transported to our centre under appropriate conditions for analysis, where we measured the levels of zinc (Zn), copper (Cu), manganese (Mn), molybdenum (Mo), vanadium (V), antimony (Sb) and chromium (Cr), and studied the levels of ICAM-1 and VCAM-1. Sample preparation was performed at the Multidisciplinary Laboratory of Yozgat Bozok University and analysis at the Science and Technology Application and Research Centre of Yozgat Bozok University. For the study, 1 mL of the blood (serum) samples was taken, and the total volume was made up to 10 mL with distilled water using 5 mL of nitric acid. The samples were stored at 4 °C until they were fed into the device. After vortexing the samples, inductively coupled plasma mass spectrometry (ICP-MS, Thermo Scientific ICAP Qc, Spectralab Scientific Inc., Buffalo, NY, USA) was used for analysis. An 11-point calibration curve was used for the analysis. Additionally, validation procedures were performed using a Seronorm WBL-2 certified sample, and the relevant method was optimised for other trace elements [22]. ICAM-1 and VCAM-1 biomarker levels were measured using an ELISA device at the Multidisciplinary Research Laboratory of the Faculty of Medicine at Bozok University in Yozgat, in accordance with the relevant kit procedure. Serum ICAM-1 and VCAM-1 levels were measured using commercially available enzyme-linked immunosorbent assay (ELISA) kits according to the manufacturer’s instructions. ICAM-1 was measured using a human ICAM-1 ELISA kit (Sandwich method; manufacturer: [Reed Biotech, Wuhan, China], catalog no: [RE2831H]), with a detection range of [31.25–2000 pg/mL]. VCAM-1 levels were determined using a human VCAM-1 ELISA kit (Sandwich method; manufacturer: [Reed Biotech, Wuhan, China], catalog no: [RE2675H]), with a detection range of [12.5–800 pg/mL]. The intra-assay coefficient of variation (CV) was <[2.8%] and the inter-assay CV was <[3.9] for both assays. Absorbance values were measured using a microplate reader, and concentrations were calculated from standard curves generated by linear regression.

The patients’ medical history with regard to kidney disease, as well as data relating to the haemodialysis procedure (e.g., dialysis frequency, efficacy and vascular access routes), was recorded. Simultaneously measured blood urea nitrogen (BUN) and creatinine levels were obtained from the hospital record system, and the glomerular filtration rate (GFR) was calculated.

### 2.3. Exclusion Criteria

The study excluded individuals with a history of acute kidney failure; those undergoing temporary haemodialysis (e.g., due to intoxication); individuals using vitamin or mineral supplements; those with renal pathologies other than atrophic kidneys evident in past radiological imaging; individuals dependent on oral nutritional solutions or intravenous nutrition; and individuals with active viral or bacterial infections. Patients who did not attend regular haemodialysis sessions and individuals with metal-containing foreign bodies in their bodies were also excluded.

### 2.4. Statistical Analysis

The data were analysed using SPSS 20.00 (Statistical Package for the Social Sciences, SPSS Inc., Chicago, IL, USA). Descriptive statistics were expressed as the mean ± standard deviation for continuous variables, and as percentages for categorical variables. The Kolmogorov–Smirnov test was used to assess normality of distribution. Independent samples *t*-tests and ANOVAs were used to compare measurements between groups for normally distributed variables, while chi-square tests were used for categorical variables. Where normal distribution was not observed, the Mann–Whitney U test and Kruskal–Wallis test were applied. A *p*-value of less than 0.01 or 0.05 was considered statistically significant. Correlation analyses were performed using the Pearson correlation test. To account for multiple testing in correlation analyses, False Discovery Rate (FDR) correction using the Benjamini–Hochberg method was applied. Adjusted *p*-values are reported where appropriate. Additionally, 95% confidence intervals (CI) were calculated for correlation coefficients to improve interpretability.

Potential confounding variables including age, sex, dialysis vintage (duration of dialysis), and major comorbidities (hypertension and diabetes mellitus) were considered in the statistical analyses. Age-adjusted analyses were performed using ANCOVA where appropriate. Dialysis vintage was evaluated using correlation analyses, while the effects of comorbidities were assessed through subgroup comparisons. Sex distribution was compared between groups and was not included as a covariate due to the absence of a significant imbalance.

## 3. Results

A total of 126 individuals were included in the study: 84 patients undergoing haemodialysis due to chronic kidney failure, and 42 healthy controls. Of these participants, 53 (39.3%) were female and 82 (60.7%) were male. Participants’ ages ranged from 18 to 65 years, with an average age of 52.43 ± 16.52 years. The mean age of the patients was 58.33 ± 16.11 years, compared to 40.64 ± 9.66 years for the healthy controls, indicating that both groups were predominantly middle-aged. When trace element levels were compared between the patient and control groups, manganese levels were significantly higher in the haemodialysis group, while zinc and chromium levels were significantly lower. Although copper, molybdenum, vanadium, and antimony levels tended to be higher in the control group, these differences were not statistically significant. These findings indicate a selective change in trace element balance in haemodialysis patients rather than a uniform change across all elements (Table 1).

When examining the effect of chronic diseases other than chronic kidney disease on trace element levels, copper (Cu) levels were found to be significantly lower in hypertensive patients (962.19 vs. 1345.14 ppb; *p* = 0.011). In diabetic patients, zinc (Zn) and manganese (Mn) levels were significantly higher in the patient group compared to the control group (Zn: 890.23 vs. 681.50 ppb, *p* = 0.048; Mn: 28.98 vs. 14.03 ppb, *p* = 0.004). No significant differences were found in other trace elements with hypertension, diabetes or coronary artery disease. Furthermore, no difference was found in trace element levels between individuals with and without a family history of kidney disease (*p* > 0.05). Correlation analyses showed that antimony levels increased with prolonged haemodialysis duration and exhibited a cumulative effect over time (*p* = 0.01). Dialysis adequacy, reflected by the Kt/V ratio, showed positive correlations with manganese, antimony, and chromium levels (*p* = 0.05), suggesting that dialysis efficiency may affect the clearance or accumulation of certain trace elements. In contrast, dialysis frequency was not found to be associated with trace element levels (*p* > 0.05) (Table 2).

**Figure 1:**
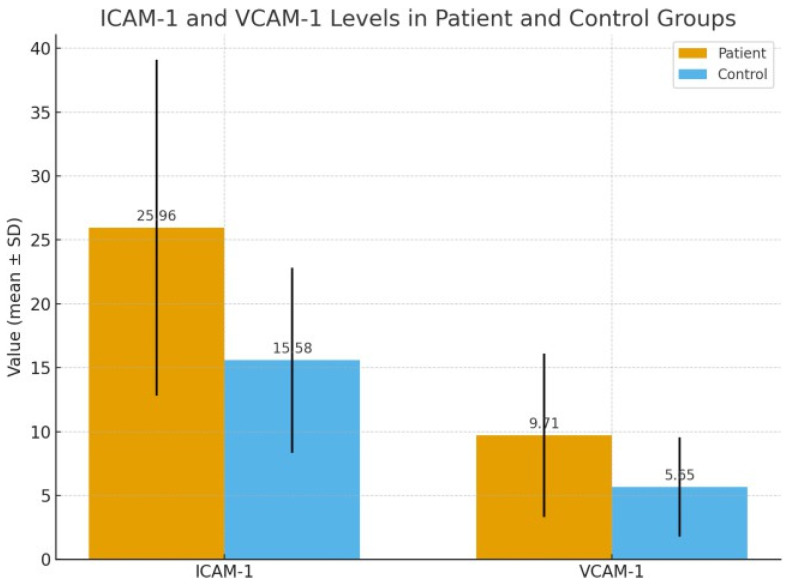
Comparison of ICAM-1 and VCAM-1 Levels Between Patient and Control Groups.

Statistical analysis was performed to examine the differences in ICAM-1 and VCAM-1 levels between the groups. The mean ICAM-1 level was 25.96 ± 13.17 ng/mL in the patient group and 15.58 ± 7.24 ng/mL in the control group. This difference was statistically significant (*p* = 0.001). Similarly, the mean VCAM-1 level was 9.71 ± 6.39 ng/mL in the patient group and 5.65 ± 3.89 ng/mL in the control group, with this difference also being statistically significant (*p* = 0.001) (Figure 1).

When the correlations between trace elements and adhesion molecules were examined, various relationships were identified. Zinc levels were inversely correlated with VCAM-1 (*p* = 0.01), while manganese levels showed a positive correlation with ICAM-1 (*p* = 0.01). Molybdenum showed a negative correlation with both ICAM-1 and VCAM-1 (*p* = 0.05 and 0.01, respectively). Additionally, antimony and chromium levels showed a positive correlation with each other (*p* = 0.01), suggesting common regulatory or exposure pathways. Zinc demonstrated a modest inverse correlation with VCAM-1 (r = −0.294, 95% CI: −0.50 to −0.07, FDR-adjusted *p* = 0.026). Manganese showed a weak to moderate positive correlation with ICAM-1 (r = 0.364, 95% CI: 0.12–0.56, FDR-adjusted *p* = 0.018). After FDR correction, the associations between Mo and ICAM-1 did not retain statistical significance (adjusted *p* > 0.05). Although certain trace elements demonstrated statistically significant correlations with adhesion molecules, the effect sizes were small to moderate, and only a subset remained significant after multiple comparison correction. These findings suggest a potential biological association but do not establish clinical significance. These findings support a potential link between trace element imbalance and endothelial activation in haemodialysis patients (Table 3).

When haemodialysis patients were examined according to the number of previous vascular interventions for fistula creation, 40 (54.8%) had undergone a single fistula intervention, 19 (26.0%) had undergone two fistula interventions, 8 (11%) had undergone three fistula interventions, and 6 (8.2%) had undergone four or more fistula interventions. (26.0%), 8 (11%) had three fistula attempts, and 6 (8.2%) had four or more fistula attempts. Ultimately, a total of 20 patients were identified whose fistulas were deemed unsuccessful, and who therefore continued haemodialysis via catheter. Of the currently active vascular access routes, 53 (63.1%) were arteriovenous fistulas and 31 (36.9%) were catheters. When the relationship between the number of fistula attempts and cellular adhesion molecules was evaluated between the groups, *p* = 0.923 was calculated for ICAM-1 and *p* = 0.091 for VCAM-1. When individuals with fistulas were divided into two groups, single vascular access and multiple vascular access, no statistically significant difference was observed between the groups in terms of ICAM-1 and VCAM-1 levels (*p* = 0.644 and *p* = 0.100, respectively). When comparing patients who were considered to have failed fistula and returned to catheter use with individuals undergoing haemodialysis with an active fistula, *p* = 0.940 was calculated for ICAM-1 and *p* = 0.538 for VCAM-1, respectively. No statistically significant differences were observed in trace element and cellular adhesion molecule levels according to the active haemodialysis vascular access type in the patient group (*p* > 0.05) (Table 4).

There was a significant age difference between the patient and control groups, additional age-adjusted analyses were performed. ANCOVA was conducted using age as a covariate. After adjustment for age, ICAM-1 and VCAM-1 levels remained significantly higher in the haemodialysis group (*p* < 0.001 and *p* = 0.001, respectively), while age itself was not a significant predictor (*p* > 0.05). In addition, age-matched subgroup analyses (±5 years) confirmed that both ICAM-1 and VCAM-1 levels were significantly elevated in haemodialysis patients compared to controls. These findings indicate that the observed differences are independent of age. Additional analyses showed that dialysis vintage and comorbidities influenced certain trace element levels but did not eliminate the observed group differences in ICAM-1 and VCAM-1.

## 4. Discussion

A comparison of trace element levels between patients undergoing haemodialysis for chronic renal failure and a control group revealed that manganese (Mn) levels were higher in the patient group and exceeded reference values (*p* = 0.019, Table 1). This suggests that monitoring haemodialysis patients for manganese accumulation may be important. The literature also reports neurological symptoms and Parkinson’s-like findings associated with manganese toxicity [25,26]. An MRI study conducted in haemodialysis and peritoneal dialysis patients showed that manganese accumulation was higher in the basal ganglia, but no clinically significant findings were detected [27]. Another study found that Mn levels were higher in haemodialysis patients and were associated with dermatological symptoms [28]. This suggests that toxicity thresholds may be at levels that cause organ damage. Regarding other trace elements, Zn, Cu, Mo, V, Sb, and Cr levels were found to be lower than in the control group; a marked decrease was observed particularly in Zn and Cr. This suggests that trace element loss may occur during the haemodialysis process. The literature recommends regular monitoring of Zn and Cu levels in haemodialysis patients [29]. Meta-analyses have also found that there is limited data on antimony (Sb) in haemodialysis patients, while Zn, Mn, and Se levels are reduced and V and Cr levels are increased [13]. Therefore, trace elements should be evaluated together for deficiency or excess. When we assessed comorbidities in patients with chronic kidney disease, we found that 54.8% had hypertension and 28.1% had diabetes mellitus. In our study, when evaluating the elemental relationship of chronic diseases, we observed that hypertension was associated with lower copper (Cu) levels, while diabetes mellitus was associated with higher zinc (Zn) and manganese (Mn) levels. Miao and colleagues identified high copper intake as a risk factor for the development of essential hypertension [30]. A cohort study conducted in China further clarified this by showing a U-shaped relationship between copper levels and hypertension, and that both high and low copper levels are risk factors for hypertension [31]. Based on our findings and the literature, we recommend evaluating copper levels in patients with resistant hypertension after haemodialysis.

A review of studies examining the relationship between manganese (Mn) and zinc (Zn) levels and diabetes mellitus has reported that diabetic nephropathy patients have lower Zn levels than healthy individuals. Low Zn levels have been observed to be a protective factor against inflammation [32]. Another study found that high Zn intake in diabetic individuals was associated with a significant reduction in the risk of atherosclerotic cardiovascular disease over a 10-year period [33]. In our study, although high Zn levels in haemodialysis patients did not reach toxic levels, this situation may provide protection; however, close monitoring is required to prevent levels from rising to inflammation thresholds. Trace elements may play a role as risk factors in chronic diseases. Metal levels should be comprehensively evaluated, particularly in diabetes mellitus and hypertension, as these constitute important aetiological factors in chronic kidney disease. It is evident that the literature on this subject is insufficient and requires further research.

Trace elements in the body can be affected by many factors in haemodialysis patients. One of these factors is nutrition. Although the amount of trace elements in the KBH diet has been considered negligible, the limited number of studies on this subject is considered a gap in the literature. In our study, the prevalence in the areas where all individuals reside within the same city (such as food content and water sources) can be minimised. In this context, it has been observed that the trace element amounts in food content and water sources in our region are within the WHO reference limits [20,21]. The chronic kidney disease patients included in our study were mostly from rural areas and were unable to adequately comply with the CKD special diet and fluid restriction due to both sociocultural and socioeconomic factors; therefore, they were unable to follow a special CKD diet. This also limited the effect of variables related to nutrition between groups. Since no special diet was applied in our study and the water consumed did not contain minerals, the effect of nutrition on our study results was considered to be limited. Furthermore, it is not expected that the parenteral treatments received by patients prior to the study would have a major effect on trace element levels absorbed by the enteral system.

In terms of haemodialysis parameters, Sb levels have increased with dialysis duration. Although Sb is at physiological levels, there may be a risk of accumulation with long-term treatment [17]. This situation may indicate that long-term clearance is insufficient. The fact that weekly dialysis frequency has no effect on trace elements supports the need for individual assessment, as increasing frequency may not be beneficial unless dialysis efficacy improves, exposure varies between individuals, and factors such as the effect of tissue accumulation should not be overlooked. When relationships with creatinine and GFR were evaluated, Mo levels increased with creatinine but decreased with GFR. This suggests that Mo may be an indicator of renal function. Mn levels decreased as GFR increased, indicating that Mn accumulation may increase with impaired renal function. The literature reports a negative correlation between creatinine levels and both copper and lithium in haemodialysis patients [34]. Similar relationships have also been reported between Zn and GFR [35]. This result can be generalised to suggest that GFR-based trace element level changes may occur in chronic kidney disease rather than haemodialysis. Finally, when examining cellular adhesion molecules, ICAM-1 and VCAM-1 levels were found to be higher in the patient group (*p* = 0.000 and 0.001), which may indicate increased cellular activity and changes in vascular structure. The literature also reports elevated ICAM-1 levels in haemodialysis patients in association with cardiovascular risk [36,37]. It has been shown that ICAM-1 levels decrease after haemodialysis, while VCAM-1 levels do not change significantly [37]. However, as our study did not examine dialysis inlet and outlet levels, we cannot comment on other parameters, but we can say that they are higher than in the healthy group and are ultimately related to vascular endothelial processes.

Intimal hyperplasia, inflammation and vascular remodelling are important pathogenic mechanisms in haemodialysis access. Inflammatory cytokines and adhesion molecules are also involved in these processes, but the direct relationship between serum levels of these molecules and fistula failure remains controversial [38]. VCAM-1 has been reported to be associated with AVF dysfunction, particularly in relation to vascular remodelling and haemodynamic parameters. For instance, one study found that VCAM-1 levels were significantly higher in patients with AVF dysfunction, and identified correlations between VCAM-1 and certain ultrasonographic parameters [39]. Another study found that soluble VCAM-1 and ICAM-1 levels were generally higher in patients with complications such as AV fistula thrombosis; however, the results were not found to have strong clinical predictive value [40]. A study involving 68 haemodialysis patients found that VCAM-1 levels were significantly higher in those with AVF dysfunction. AVF dysfunction in this study was defined by brachial artery blood flow and vascular resistance, and VCAM-1 levels were shown to be significantly higher in this group [39]. However, our study found no statistically significant relationship between arteriovenous fistula failure and cellular adhesion molecules (ICAM-1 and VCAM-1). However, the heterogeneity of the underlying causes of fistula failure in patients with repeated attempts at fistula creation may have contributed to this result. In particular, the inability to distinguish clearly the extent to which fistula failure is due to primary vascular damage versus non-vascular factors, such as surgical technique, haemodynamic factors, thrombotic events or infectious processes, has made it difficult to establish a relationship with cellular adhesion molecules. Considering that these molecules are indicators of endothelial dysfunction and vascular inflammation, a classification based solely on the presence of fistula failure may be insufficient to reflect their relationship with these biomarkers. To make a more accurate assessment, prospective studies with larger sample sizes are needed to identify cases where fistula failure developed in isolation due to vascular damage. Where possible, these studies should be supported by imaging and histopathological data. Such studies may reveal the potential role of cellular adhesion molecules in fistula failure more clearly.

Examining the relationships between trace element adhesion molecules reveals that manganese has dual effects on endothelial cell function. At low or optimal levels, manganese (Mn) may protect the endothelium by supporting antioxidant enzyme activity; however, excessive exposure may trigger oxidative stress and inflammation. One study found that external Mn supplementation reduced ICAM-1 expression and decreased endothelial cell adhesion, suggesting a protective role for Mn in the endothelium [41]. However, another study associated high Mn levels with oxidative stress-mediated endothelial dysfunction [42]. This suggests that excessive Mn exposure may trigger an inflammatory response in endothelial cells. The literature also reports that high Mn concentrations increase NF-κB activation via oxidative stress, which may elevate the expression of adhesion molecules such as ICAM-1 [43]. These results support the potential adverse effects of Mn toxicity on vascular inflammation. Our findings provide evidence that Mn at toxic levels may trigger inflammation, but further studies are needed to fully elucidate the mechanism. Although several statistically significant correlations were observed, most of them demonstrated small effect sizes (r < 0.30). According to conventional interpretations, such correlations indicate weak associations and should not be considered strong predictors. After correction for multiple comparisons using the false discovery rate approach, only a subset of these associations remained statistically significant. Therefore, the observed relationships should be interpreted as hypothesis generating rather than clinically definitive. Larger prospective studies are required to determine whether these associations have meaningful clinical implications. The literature reports that increased Zn levels may be associated with increased ICAM-1 and VCAM-1, which may be linked to endothelial dysfunction [44]. The literature suggests that low serum zinc may increase ICAM-1 and VCAM-1 in endothelial cells, thereby increasing the endothelial inflammatory response. Furthermore, environmental and experimental studies have demonstrated that various metals can have adverse effects on the vascular endothelium [45,46,47]. When these findings are considered together, it is evident that trace elements have complex and multifaceted relationships with vascular endothelial function; however, further studies are needed to clarify the clinical significance of these relationships. In our study, the increased vascular endothelial adhesion molecules observed in patients undergoing haemodialysis due to chronic renal failure may be related to trace element levels; however, these findings do not prove a causal relationship, and further research is needed.

There was a significant age difference between the haemodialysis and control groups, age was considered a potential confounder for both trace element levels and endothelial adhesion molecules. However, age-adjusted analyses using ANCOVA demonstrated that the elevated ICAM-1 and VCAM-1 levels observed in haemodialysis patients remained statistically significant after adjustment for age, whereas age itself was not a significant predictor. Furthermore, age-matched subgroup analyses yielded consistent results. These findings indicate that the increased expression of cellular adhesion molecules in haemodialysis patients is primarily related to the pathophysiological effects of chronic kidney disease and the haemodialysis process rather than chronological ageing alone. This observation is clinically relevant, as ageing is known to be associated with low-grade endothelial activation; however, our results suggest that the magnitude of endothelial dysfunction in haemodialysis patients exceeds that expected from ageing alone. Uremic toxins, oxidative stress, chronic inflammation, and trace element imbalance may collectively contribute to the upregulation of ICAM-1 and VCAM-1 in this population. These results indicate that the observed differences in endothelial adhesion molecules are independent of age and are more likely related to disease-specific and dialysis-related mechanisms.

This study has certain limitations. Firstly, as cases of chronic kidney disease (CKD) not undergoing dialysis were not included in the study, the findings are only valid for haemodialysis patients and healthy controls, and the isolated effects of CKD could not be evaluated. Studies covering all stages of CKD could more clearly reveal the effects of the disease alone on trace elements. Furthermore, trace element levels were measured only in serum and may not reflect tissue accumulation or long-term exposure. The absence of pre- and post-dialysis measurements also prevented the evaluation of the acute effects of haemodialysis. Considering these limitations, the results should be interpreted with caution, and more comprehensive methodological approaches should be adopted in future studies.

## 5. Conclusions

Our study is a clinical trial investigating trace element levels in haemodialysis patients, as well as the relationship between resident adhesion molecules and trace elements. Disturbances in trace element homeostasis and significant increases in cellular adhesion molecule levels (ICAM-1 and VCAM-1) have been observed in haemodialysis patients. Dialysis adequacy and cumulative dialysis time were found to be associated with certain trace elements, whereas vascular access type and fistula history did not show a significant effect. The fact that ICAM-1 and VCAM-1 levels are higher in haemodialysis patients than in controls, independent of age, and correlate with certain trace elements, points to a possible link between trace element imbalance and endothelial activation The absence of a relationship between fistula failure and the number of previous AVF interventions and adhesion molecule levels suggests that fistula failure is influenced by multiple factors, not solely by proven vascular endothelial causes. This study has certain limitations. Firstly, as cases of chronic kidney disease (CKD) not undergoing dialysis were not included in the study, the findings are only valid for haemodialysis patients and healthy controls, and the isolated effects of CKD could not be evaluated. Studies covering all stages of CKD could more clearly reveal the effects of the disease alone on trace elements. Furthermore, trace element levels were measured only in serum and may not reflect tissue accumulation or long-term exposure. The absence of pre- and post-dialysis measurements also prevented the evaluation of the acute effects of haemodialysis. Considering these limitations, the results should be interpreted with caution, and more comprehensive methodological approaches should be adopted in future studies.

## Figures and Tables

**Table 1:** Levels of Trace Elements with reference ranges for metals according to ACGIH [23]-Except Vanadium [24].

Unit (ppb)	Patient (n:84)		Control (n:42)		*p*	Limit (ppb)
	Mean	STD	Mean	STD		
**Zn**	795.87	555.78	1015.14	361.49	0.018 *	666–1100
**Cu**	1275.44	574.61	1404.35	658.61	0.244	850–1900
**Mn**	22.79	27.31	12.87	8.25	0.019 *	4.7–18.3
**Mo**	0.60	1.06	0.66	1.01	0.760	0.3–2
**V**	2.66	1.51	3.07	1.65	0.150	0.032–0.095
**Sb**	1.51	0.64	1.56	0.68	0.695	0–3
**Cr**	2.88	1.29	3.65	1.98	0.007 *	0.7–28

[Zn (zinc), Cu (copper), Mn (manganese), Mo (molybdenum), V (vanadium), Sb (antimony), Cr (chromium)]; all unit (ppb = ng/mL); *p*-values. * represent significance at 5%.

**Table 2:** Relationship between Dialysis Adequacy Indices, Duration and Frequency, and Pre- and Post-Dialysis Renal Function Indicators and Trace Element Levels.

	Zn	Cu	Mn	Mo	V	Sb	Cr
**Duration of dialysis**	0.114	−0.071	0.235 *	−0.085	−0.038	0.295 **	0.204
**Frequency of dialysis**	0.057	0.074	−0.093	0.04	−0.132	−0.052	−0.125
**Kt/V**	0.075	0.134	0.256 *	−0.142	−0.09	0.272 *	0.259 *
**BUN pre-dialysis**	0.011	−0.112	0.161	0.121	0.096	0.109	−0.057
**BUN post-dialysis**	−0.026	−0.047	0.092	0.252 *	0.139	−0.025	−0.134
**CRE pre-dialysis**	−0.02	−0.117	0.182	0.230 *	−0.044	0.074	0.014
**CRE post-dialysis**	−0.053	−0.111	0.078	0.281 **	0.083	−0.023	−0.104
**GFR pre-dialysis**	−0.054	0.131	−0.226 *	−0.189	0.074	−0.038	−0.023
**GFR post-dialysis**	0.002	0.159	−0.178	−0.294 **	−0.016	0.058	0.105

[Zn (zinc), Cu (copper), Mn (manganese), Mo (molybdenum), V (vanadium), Sb (antimony), Cr (chromium), BUN (Blood Urea Nitrojen), CRE (Creatinin), GFR (Glomerular filtration rate)]; *p*-values. * and ** represent significance at 5%, and 1%.

**Table 3:** Correlation Between Trace Element Levels and Adhesion Molecules.

	Cu	Mn	Mo	V	Sb	Cr	ICAM-1	VCAM-1
**Zn**	0.12	0.164	−0.022	−0.088	0.291 **	0.419 **	0.117	−0.294 **
**Cu**	1	−0.082	0.128	−0.105	0.001	0.072	0.074	−0.06
**Mn**		1	−0.094	−0.062	0.418 **	0.162	0.364 **	0.056
**Mo**			1	0.032	−0.191 *	−0.073	−0.205 *	−0.241 **
**V**				1	−0.203 *	−0.112	−0.183 *	0.051
**Sb**					1	0.252 **	0.056	−0.016
**Cr**						1	−0.094	−0.132
**ICAM-** **1**							1	0.165

[Zn (zinc), Cu (copper), Mn (manganese), Mo (molybdenum), V (vanadium), Sb (antimony), and Cr (chromium)]; *p*-values. * and ** represent significance at 5%, and 1%.

**Table 4:** Evaluation of Trace Elements and Adhesion Molecules According to Vascular Access Type.

	Fistula		Catheter		*p*
Unit (ppb)	Mean	STD	Mean	STD	
**Zn**	833.58	682.16	751.01	269.12	0.497
**Cu**	1326.88	620.95	1209.197	498.27	0.327
**Mn**	25.32	31.22	18.39	19.84	0.204
**Mo**	0.63	1.20	0.51	0.80	0.558
**V**	2.75	1.76	2.43	0.95	0.269
**Sb**	1.58	0.68	1.39	0.57	0.173
**Cr**	2.82	1.39	2.94	1.13	0.660
**ICAM_1**	25.72	13.36	26.64	13.08	0.748
**VCAM_1**	10.18	6.05	9.19	6.16	0.471

[Zn (zinc), Cu (copper), Mn (manganese), Mo (molybdenum), V (vanadium), Sb (antimony), and Cr (chromium)].

## Data Availability

The data sets used during the current study are available from the corresponding author on reasonable request.
